# Advanced Sensing and Control for Connected and Automated Vehicles

**DOI:** 10.3390/s22041538

**Published:** 2022-02-16

**Authors:** Chao Huang, Haiping Du, Wanzhong Zhao, Yifan Zhao, Fuwu Yan, Chen Lv

**Affiliations:** 1 Department of Industrial and Systems Engineering, The Hong Kong Polytechnic University, Hong Kong 999077, China; 2 Faulty of Engineering and Information Science, University of Wollongong, Wollongong, NSW 2522, Australia; hdu@uow.edu.au; 3 Department of Vehicle Engineering, Nanjing University of Aeronautics & Astronautics, Nanjing 213300, China; zwz@nuaa.edu.cn; 4 School of Aerospace, Transport and Manufacturing, Cranfield University, Bedford MK43 0AL, UK; yifan.zhao@cranfield.ac.uk; 5 Hubei Key Laboratory of Advanced Technology for Automotive Components, Wuhan University of Technology, Wuhan 430070, China; yanfw@whut.edu.cn; 6 School of Mechanical and Aerospace Engineering, Nanyang Technological University, Singapore 639798, Singapore; lyuchen@ntu.edu.sg

## About the Editors

In recent years, connected and automated vehicles (CAV) have been a transformative technology that is expected to reduce emissions and change and improve the safety and efficiency of the mobilities. As the main functional components of CAVs, advanced sensing technologies and control algorithms, which gather environmental information, process data, and control vehicle motion, are of great importance. The development of novel sensing technologies for CAVs has become a hot spot in recent years. Thanks to the improved sensing technologies, CAVs are able to interpret sensory information to further detect obstacles, localize their positions, and navigate themselves and interact with other surrounding vehicles in the dynamic environment. Furthermore, leveraging computer vision and other sensing methods, in-cabin human body activities, facial emotions, and even mental states can also be recognized.

This Special Issue of *Sensors* aims at reporting on some of the recent research efforts on this increasingly important topic. The 12 accepted papers in this Issue cover vehicle position estimation [1], vehicle dynamic parameters estimation [2], cooperative collision warning systems [3], small object detection [4], impact identification of the driver’s driving performance on executive control function [5], hybrid path planning for autonomous driving [6], trajectory tracking for autonomous driving [7], vehicle stability control [8], vehicle stability and ride comfort control [9], urban platooning protocol design for platoon [10], path planning algorithm for platooning [11], and self-driving architecture design for CAV platoon [12].

In the next paragraphs, a brief description of the content of each contribution forming the Special Issue is provided.

In [1], a data-driven object vehicle estimation scheme to solve measurement uncertainty and latency problems in radar systems is proposed. An accuracy model considers the different error characteristics depending on the zone. The accuracy model was used to solve the measurement uncertainty of radar. The authors also develop latency coordination for the radar system by analyzing the position error depending on the relative velocity. The authors claimed that proposed estimation method produces improved performance over the conventional radar estimation and previous methods.

In [2], a two-stage estimation method, consisting of multiple-models and the Unscented Kalman Filter, is proposed to estimate vehicle dynamic parameters. During the first stage, the longitudinal vehicle dynamics model is used. Through vehicle acceleration/deceleration, this model can be used to estimate the distance between the vehicle centroid and vehicle front, the height of vehicle centroid, and tire longitudinal stiffness. The estimated parameter can be used in the second stage. During the second stage, a single-track vehicle model with roll dynamics is adopted. By making the vehicle have continuous steering, this vehicle model can be used to estimate tire cornering stiffness, the vehicle moment of inertia around the yaw axis, and the moment of inertia around the longitudinal axis. The results in [2] show that the proposed method is effective and vehicle dynamic parameters can be well estimated.

A vehicle-to-vehicle (V2V) cooperative collision warning system (CCWS) consisting of an ultra-wideband (UWB) relative positioning/directing module and a dead reckoning (DR) module with wheel-speed sensors is proposed in [3]. An over-constrained localization method is proposed to calculate the relative position and orientation with the UWB data more accurately. Vehicle velocities and yaw rates are measured by wheel-speed sensors. An extended Kalman filter (EKF) is applied based on the relative kinematic model to combine the UWB and DR data. Finally, the time to collision (TTC) is estimated based on the predicted vehicle collision position. The authors of [3] concluded that the proposed method significantly improves the positioning and directing, and the proposed system can efficiently provide collision warning.

As small object detection is very important for the understanding of traffic scene environments, [4] proposes a small object detection method in traffic scenes based on attention feature fusion. First, a multi-scale channel attention block (MS-CAB) is designed, which uses local and global scales to aggregate the effective information of the feature maps. Based on this block, an attention feature fusion block (AFFB) is proposed, which can better integrate contextual information from different layers. Finally, the AFFB is used to replace the linear fusion module in the object detection network and obtain the final network structure. The authors in [4] conclude that the proposed approach increases the mAP of all objects by 0.9 percentage points on the validation set of the traffic scene dataset BDD100K, and at the same time, increases the mAP of small objects by 3.5%.

To explore the relationship between the driver’s driving performance and executive control function, the authors of [5] invite a total of 35 healthy subjects to take part in a simulated driving experiment and a task-cuing experiment. The subjects were divided into three groups according to their driving performance (aberrant driving behaviors, including lapses and errors) by the clustering method. Then, the performance efficiency and electroencephalogram (EEG) data acquired in the task-cueing experiment were compared among the three groups. The authors concluded that this research presented evidence of the close relationship between executive control functions and driving performance.

In [6], a hybrid path planning is proposed to avoid unsatisfying path generation and to improve the performance of autonomous driving by combining the potential field with the sigmoid curve. The repulsive and attractive potential fields are redesigned by considering the safety and the feasibility. Based on the objective of the shortest path generation, the optimized trajectory is obtained to improve the vehicle stability and driving safety by considering the constraints of collision avoidance and vehicle dynamics. The effectiveness is examined by simulations in multiobstacle dynamic and static scenarios. The authors claimed that the proposed method shows better performance on vehicle stability and ride comfortability than that of the traditional potential field-based method in all the examined scenarios during autonomous driving.

Trajectory tracking is a key technology for precisely controlling autonomous vehicles. A trajectory-tracking method based on model predictive control is proposed in [7]. Instead of using the forward Euler integration method, the backward Euler integration method is used to establish the predictive model. To meet the real-time requirement, a constraint is imposed on the control law, and the warm-start technique is employed. The authors of [7] concluded that the proposed the tracking performance of the proposed controller is much better than that of controllers using the forward Euler method.

In [8], studies on comprehensive three-dimensional vehicle dynamics modelling and stability control strategies in the event of a sudden tire blow-out are conducted. An integrated control framework for a combined yaw plane and roll-plane stability control is presented. The authors of [8] concluded that the proposed lower-level MPC can successfully improve the roll stability in the challenging scenario of a tire blow-out during a fishhook maneuver when the vehicle has a big load transfer.

A Unified Chassis Control (UCC) strategy for enhancing vehicle stability and ride comfort by the coordination of four In-Wheel Drive (IWD), Four-Wheel Independent Steering (4WIS), and Active Suspension Systems (ASS) is designed in [9]. A hierarchical control structure was adopted to realize the UCC, including high-level sliding mode control, fixed point CA, and a normal tire force robust tracking controller. The authors claimed that the proposed method can effectively realize the tire force distribution to control the vehicle body attitude and driving stability even in high-demanding scenarios.

When an existing vehicle platoon is applied to urban roads, many challenges are more complicated to address than highways. They include complex topology, various routes, traffic signals, intersections, frequent lane changes, and communication interference depending on a higher vehicle density. To address these challenges, [10] propose a distributed urban platooning protocol (DUPP) that enables high mobility and maximizes flexibility for driving vehicles to conduct urban platooning in a decentralized manner. DUPP performs forwarder selection using an analytic hierarchy process. The performance of the proposed DUPP is compared with that of ENSEMBLE, which is the latest European platooning project. The authors of [10] concluded that the proposed DUPP is well suited to dynamic urban environments by maintaining a vehicle platoon as stable as possible.

In [11], a path planning algorithm for the platooning of articulated cargo trucks has been developed. Using the Kalman filter, V2V communication, and a novel update-and-conversion method, each following vehicle can accurately compute the trajectory of the leading vehicle’s front part for using it as a target path. The authors claimed that on severe driving scenarios, the proposed algorithm could provide lateral string stability and robustness for truck platooning.

A self-driving architecture combining the sensing, planning, and control for CAV platoons in an end-to-end fashion is proposed in [12]. This multi-task model can switch between two tasks to drive either the leading or following vehicle in the platoon. The architecture is based on an end-to-end deep learning approach and predicts the control commands, i.e., steering and throttle/brake, with a single neural network. The inputs for this network are images from a front-facing camera, enhanced by information transmitted via V2V communication. The authors claimed that the approach eliminates casual confusion for the following vehicle, which is a known limitation of end-to-end self-driving.

In summary, there is a huge potential for CAV in collision avoidance, safety improvement and driving stability improvement. The papers gathered in this Special Issue contributed by proposing solutions to the general problem of state and/or parameter estimation [1,2,3], obstacle detection [4], driver behavior estimation [5], and/or classical academic problems such as path planning [6], path tracking [7], and stability control for autonomous driving [8,9] and/or by suggesting applications of the combined use of sensors and advanced algorithms to platooning [10,11,12], thus showing the theoretical challenges and practical interest of this research topic. Finally, we wish to thank the authors, reviewers, and journal staff for their commitment and effort, which made it possible to complete this Special Issue on time.
